# Patients´ Variations of Reflection About and Understanding of Long-Term Illness- Impact of Illness Perception on Trust in Oneself or Others

**DOI:** 10.2174/1874434601711010043

**Published:** 2017-04-17

**Authors:** Håkan Nunstedt, Gudrun Rudolfsson, Pia Alsen, Sandra Pennbrant

**Affiliations:** University West, Department of Health Sciences, Trollhattan, Sweden

**Keywords:** Illness perception, Knowledge, Learning, Primary healthcare, Reflection, Understanding

## Abstract

**Background:**

Patients' understanding of their illness is of great importance for recovery. Lacking understanding of the illness is linked with the patients' level of reflection about and interest in understanding their illness.

**Objective:**

To describe patients’ variations of reflection about and understanding of their illness and how this understanding affects their trust in themselves or others.

**Method:**

The study is based on the “Illness perception” model. Latent content analysis was used for the data analysis. Individual, semi-structured, open-ended and face-to-face interviews were conducted with patients (n=11) suffering from a long-term illness diagnosed at least six months prior to the interview. Data collection took place in the three primary healthcare centres treating the participants.

**Results:**

The results show variations in the degree of reflection about illness. Patients search for deeper understanding of the illness for causal explanations, compare different perspectives for preventing complication of their illness, trust healthcare providers, and develop own strategies to manage life.

**Conclusion:**

Whereas some patients search for deeper understanding of their illness, other patients are less reflective and feel they can manage the illness without further understanding. Patients' understanding of their illness is related to their degree of trust in themselves or others. Patients whose illness poses an existential threat are more likely to reflect more about their illness and what treatment methods are available.

## INTRODUCTION

The patient's needs for knowledge and understanding about their illness are of great importance for the patient's self-care and recovery process [1-3]. The understanding the patient has about their illness may also be an important prerequisite for adherence and participation. Depending on what diagnosis the patient has, the patient's desire to know more about their illness varies. One study found that almost a quarter of the patients had received insufficient information about their illness, self-care methods and the significance of the medication [4]. Patients often have insufficient knowledge of disease processes; want to know more about the course of their illness, and show great variations in the need for knowledge; which is at its greatest immediately following diagnosis and during relapse [5, 6]. Studies also show that patients do not understand the illness despite receiving information from and being in close contact with a specialist [7]. Several studies have underlined the importance of providing individualised information adapted to the patient's age and ability to understand [8-11]. Doing so can be crucial for how patients perceive, accept and understand their illness when receiving their medical diagnosis. One should encourage patients to be well-informed and to act proactively with the support of their healthcare actors [12].

### Perception of One’s Own Illness

There is a difference between the patient's perception of their illness and the medical expression of this perception. The difference is often described in terms of illness versus disease, where 'illness' is the patient's feeling and experience of illness and 'disease' is the medical diagnosis and explanation of the illness. The distinction between illness and disease is a theoretical model which does not necessarily reflect the care and the complexity of the meeting between the patient and the healthcare provider. The patient's self-perceived state of health (illness) and the medical explanation of the disorder (disease) should be considered as a whole as the understanding of the one influences the understanding of the other [13]. Patients often interpret their experiences of bodily changes in terms of medical explanations even before they consult a doctor. According to Leventhal *et al.* [14], an individual's perception of factors related to their illness or symptoms influence their coping behavior. Moreover, a patient's illness perception is not static; it changes over time depending on contextual, cultural and relationship factors [15]. Illness experiences and perceptions are culturally shaped by the way the person has learned to act and think [13] and the meaning of the symptoms and illness varies depending on the patient's ethnic and cultural background [16]. Furthermore, a person's culturally determined beliefs about the cause of the illness will influence their health-related behavior [17]. Illness experiences and perceptions are also connected with biopsychosocial processes which affect the patient's ability to perceive and understand illness symptoms and to take effective action to improve their health [18].

To understand the patient's illness perception is central to healthcare providers in order to evaluate and support the patient's individual understanding of their illness and to support the patient's empowerment and self-care ability. This requires that healthcare providers see the patient's understanding of their illness as a basic and necessary resource in the care [19]. Although patients often search the Internet for information about their symptoms or illness, such 'objective' information does not always coincide with their individual understanding of the symptoms or illness. In these situations, it is important for healthcare providers to use the patient's illness perception as an additional resource when helping the patient create their individual illness perception [20].

The patient's perceptions of their health and illness are based on their individual life-world perspective. A person's 'life-world' consists of, among other things, their memories, everyday experiences, and expectations for the future. This means that one cannot draw an objective picture of the patient; instead one must focus on the patient's world as they actually perceive and live it [21]. The patient's illness perspective represents their attitudes, beliefs, experiences and perceptions of what it means to be a person with an illness in a particular context. A patient's social circumstances and the consequences of the diagnosis on their personal sphere affect how they perceive and react to the illness [13]. The patient's understanding of their illness is therefore an important factor for the planning of meaningful healthcare [8].

### Illness Perception Model

The illness perception model [22] illustrates how patients' illness reasoning differs in relation to the degree of trust they place in their own judgment (*trust in oneself*) or in other people (*trust in others*). *Trust in oneself* refers to patients who have trust in themselves and believe that they can control their illness and prevent it from becoming worse. These patients are more involved in their own healthcare, search information about their illness, and can self-manage their lives. *Trust in others* refers to patients who place their trust in the ability and judgment of others and have less confidence in their own ability to control the illness. These patients do not actively search more information about the illness, are less motivated to improve their own health situation, and need more help from others, such as healthcare providers and relatives.

**Fig. (1):**
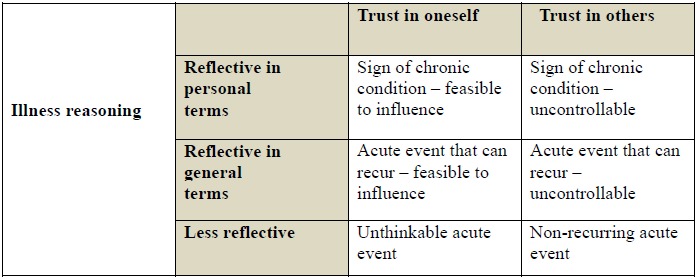
Conceptual model describing variations in illness perception related to the *illness reasoning* of myocardial infarction patients (Alsén *et al*., 2008).

Illness reasoning comprises three dimensions with variations in terms of the patient's reflection about their illness. Patients thinking in personal terms reflect more carefully and deeply about their illness; they want deeper understanding of the illness and search for causal explanations. Patients reflecting and thinking in general terms think more about the possible underlying causes and impact of their illness; they think in terms of preventing complications and are satisfied with a more general and not particularly detailed understanding of their situation. Some patients are less reflective and have no particular thoughts about their illness; their thoughts are more superficial and sporadic, especially when the illness symptoms do not affect them, and their ideas are more predetermined and difficult to influence [22]. In their study of the illness perception of myocardial infarction patients, Alsén *et al*. [22] identified six variations of illness perceptions (Fig. **1**).

### Aim

The aim of the study was to describe patients’ variations of reflection about and understanding of their illness and how this understanding affects their trust in themselves or others.

## MATERIAL AND METHODS

### Design

A qualitative design was used to describe patients’ variations of reflection about and understanding of their illness and how this understanding affects their trust in themselves or others. The study was based on individual interviews using latent content analysis [23, 24]. This qualitative design allows to study and understand a person's subjective understanding, thoughts, processes and experiences by allowing the person to express them in their own words. This type of design is appropriate when existing research on a phenomenon is limited [25], which is why the researchers found it promising for gaining insight into patients’ reflection about and understanding of their illness.

### Participants

The participants were patients (N=11) suffering from a long-term illness that had been diagnosed at least six months prior to the time of the interview. The patients’ diagnoses concerned one or more of the following conditions: arthrosis, atrial fibrillation, chronic pain, hypertension, hyperthyroidism, obstructive lung disease and type 2 diabetes. The interview inclusion criteria were: the patient must be at least 18 years of age, be capable of speaking and understanding Swedish, and have received treatment in primary healthcare for at least six months. The patients’ sociodemographic variables are shown in Table **1**.

### Data Collection

The participants were selected by healthcare providers at three primary healthcare centres in western Sweden. The healthcare professionals informed their patients about the study's purpose and invited them to take part in it. Consenting patients were contacted by telephone, either by the healthcare professional or by an interviewer, to schedule a time for the interview. Data collection took place from June 2013 to March 2014 through individual face-to-face interviews held in the three primary healthcare centres where the participants were being treated. The interview questions were semi-structured and open-ended, and the interview guide called for interviewers to ask additional questions depending on the respondents’ answers. The questions asked concerned, for instance, what understanding the respondents had about their illness, if they thought it was important for them to have understanding of their illness, or if they wanted better understanding of their illness. The interviews lasted between 28 and 75 minutes (mean 47 minutes).

### Ethical Considerations

Approval for the study was obtained from the Regional Board of Ethics at the University of Gothenburg (Dnr 873-12). Participating patients were told that participation was entirely voluntary, and that they were free to withdraw, with no reason required, from the interview and the study at their leisure. Each patient provided written informed consent before the interview was carried out.

### Data Analysis

The data were initially analysed inductively to identify variations of reflection about and understanding of long-term illness. The data were then sorted deductively and placed in the Illness reasoning model. The interview texts were analysed by means of qualitative content analysis using the latent variables interpreted from the text as the basis of the analysis [23, 24]. Initially, the entire recorded interview text was read repeatedly to achieve an overall understanding. In the next step, the individual texts were broken down according to their meaning-bearing units relating to the study's aim. The identified meaning units were condensed to their most significant parts. The underlying messages of the condensed meaning units were then interpreted and coded. The codes were continually adjusted to make the inductive process more rigorous [25]. The patterns found during the analysis and the coded units were sorted and subthemes were identified, which were categorised together into three main themes describing the latent meaning of the overt statements [23, 24, 25]. The transcribed interviews were reviewed by all of the researchers. After nine interviews had been conducted, the researchers together assessed whether or not the data was saturated in relation to the study’s aim. Following this first assessment, two additional interviews were conducted and a second data saturation assessment was conducted and concluded that no further interviews were needed. According to Cooper and Endicott [26] between five and eight participants is usually sufficient to achieve data saturation. After the last two interviews, the researchers concluded that the material was rich in qualitative statements and corresponded to the study’s aim, and that further interviews were unlikely to add any major patterns or variations to the gathered material. Table 2 provides a description of the implemented analysis process.

### Trustworthiness

Trustworthiness can be defined as credibility, dependability, confirmability and transferability. When evaluating qualitative data, these issues must be considered [27]. In this study, credibility [25] was achieved by carrying out individual interviews choosing participants from various healthcare centres and with various illness problems and by letting participants describe how they understand their illness and how it affected their management of life. To provide a broader picture of the problem, participants with several illnesses and with rich experiences of their illness were included in the study. Credibility [28] was also achieved by conducting individual interviews in which the participants described how they understood their illness. The procedure for data analysis and creation of themes and subthemes has been described above. The analysis process was characterised by critical review by all researchers. The researchers also read all of the interviews and jointly defined the themes and subthemes to ensure the study’s dependability. Confirmability [28] was achieved in the course of the analytical process by comparing the codes and subthemes with the interviews throughout the analytical process. Confirmability was also strengthened by relating our results to earlier research and by deductively sorting and placing the result in the Illness reasoning model. The unique answers of the participants and the inductive process sustain the study's confirmability. Furthermore, to meet the requirements for peer debriefing [27], in the course of the analytical process, the authors read the interviews, checking the coding of the condensed meaning units, and jointly discussed both the analysis and the results. For the sake of trustworthiness, clear descriptions of the interviewed patients' backgrounds and of the method for finding and condensing meaning units have been provided. Furthermore, examples from the interviews have been presented so that readers may judge whether this study's findings can be transferred to other settings. 

## RESULTS

The study participants ranged in age from 49 to 81 and were suffering from a long-term illness that had been diagnosed at least six months prior to the time of the interview. Themes and subthemes describe patients’ variations of reflection about and understanding of their long-term illness. Three themes with subthemes were identified: *patients actively seek knowledge of the illness for causal explanations*, *patients compare different perspectives to get knowledge for preventing complication of their illness* and *patients have trust in healthcare providers’ knowledge and develop strategies to manage life*.

### Patients Actively Seek Knowledge of the Illness for Causal Explanations

When the first symptoms appeared and before the illness was diagnosed, patients tried to find out what had caused the illness and search for explanations for deeper understanding. Subsequently, despite having been informed by healthcare providers about the illness, the patients felt an additional need for deeper understanding about the illness and therefore continued to search on their own.

#### Patients Search for Deeper Understanding About Their Illness Through Various Sources of Information

Following receipt of a diagnosis and information about it, patients felt a need for more understanding of their illness. Patients therefore searched the Internet using the diagnostic name as a search keyword. Patients also searched in other sources such as magazines, brochures or, in some cases, research articles that dealt with their own illness.

I've been online and read some articles … about various benchmarks and stuff … I tend to be well-read and always ask about my [test] values … so I have some control myself.

Some patients were particularly active and searched the Internet for their first signs and for different possible diagnoses. Sometimes, the patient's first Internet search led to the worst possible cause of explanation for their illness.

I didn't want to speculate about something I knew nothing about … One tends to think of the worst-case scenario … So I thought I should do an Internet search.

#### Patients Try to Understand the Relationship Between Root Cause, Trigger Factor and Present State

Patients felt a need to understand the connection between the root cause of their illness, its triggering factor, and their present state of health. Patients combined a clearly stated belief about what caused their illness with an active search to identify the root cause. In some cases, patients linked their signs of illness with a diagnosis and presented their thoughts during their medical consultations. Patients also asked relatives whether or not the family had a history of the possible illness, which in the affirmative could explain the symptoms by reason of possible heredity.

It's in my family background. My mother got her joint problems when she was between forty and fifty years old.

Sometimes, the patients speculated about the cause triggering the illness. In some cases, the diagnosis received from a healthcare provider came as a relief, as it proved to be less serious than the patient had initially feared.

#### Patients Want Clear Information from Healthcare Providers to Better Understand the Illness

The information received from healthcare providers was important for the patient as a support to better understand their illness, especially when the healthcare providers took time to provide more detailed information. Sometimes, healthcare providers did not have time to provide adequate information about the illness or other factors affecting the patient's medical condition.

Most of the information that I received came from the physiotherapist. Doctors don't have much time to talk about the illness. They investigate and write out prescriptions.

Sometimes, patients received a diagnosis, but no further information about its meaning. Consequently, they wished that healthcare providers would take more time to provide information on a more understandable level. The patients also wanted to have an opportunity to ask questions when they were uncertain about what the healthcare provider had actually said.

No one at the healthcare centre described the illness … I can only say that I have type 2 diabetes.

### Patients Compare Different Perspectives to Get Knowledge for Preventing Complication of Their Illness

Patients used different perspectives as references for possible underlying causes of their illness to prevent complications. The sources consisted of data on sampling, drug effects or experiences from people with the same or similar illnesses.

#### Patients Compare Test Results and Reported Medication Effects Without Understanding the Reference Values

Patients often compared their test results with reference values or described drug effects, noting that a test value was high or low, but not being capable of relating the value, and its consequences, to their illness situation.

They took long-term tests and my results have been 4.9-5 the last three times, which is very low actually.

Sometimes, patients described drug effects in line with the healthcare provider predictions, but without really understanding their implication. Similarly, patients often remembered the name of a drug mentioned by the healthcare provider, but had not always understood the meaning of the information provided about the drug.

#### Patients use Experiences and Information from Other Peoples’ Illness Situations and Compare them with their own Situation

Patients sought experiences from people suffering from the same or similar illnesses and compared the information obtained with their own illness and life situation. One patient had created a website dedicated to the illness and built up a large online social network of people with similar problems. This network served both an informational and a confirmatory purpose as the patient and the website visitors used its discussion forum to share information and comments about healthcare providers, treatments and life situations.

We are a group of people who have problems with pain that medications cannot alleviate; we are disabled in the sense that we cannot move about without feeling pain and doctors usually cannot do anything about it.

### Patients have Trust in Healthcare Providers’ Knowledge and Develop Strategies to Manage Life

As patients trusted the healthcare providers, and counted on such providers to have the right information needed for treatment and self-care measures, patients developed strategies to deal with their life situation and to endure without needing more understanding of the illness.

#### Patients Accept and Manage Life

After receiving a diagnosis of their illness, patients accepted the illness and developed strategies to manage or distance themselves from it in order not to let it affect their lives too much. The strategies enabled them to manage their illness and to have a good life despite its effects. Patients had no need to reflect more about the illness and its manifestations, and fought against the pain and became tougher when positive effects of medication or other treatment failed to occur.

If physical therapy doesn't help, then one can try cortisone; if that doesn't work, then one can operate and replace the knee; but I'll keep resisting it for as long as possible.

In some cases, the patient was very physically active and refused to be hindered by pain or other symptoms. In some cases, physical activities helped patients temporarily forget their illness and pain. One patient described his reasoning when helping a neighbour with roofing work.

It's not that I'm afraid of heights, but I'm stiff and cannot move about freely … It [the roof] is uneven so one has move about in strange ways, making the joints hurt … But this doesn't keep me from doing work on the roof from time to time.

Patients did not allow the illness to bother them too much in their daily life and strove to maintain a good quality of life. To this end, patients changed their perspective on the illness, seeing it more as a wound that would heal than as a debilitating condition. In some cases, patients had not received any direct information or instructions about the illness and did not feel any need therefore, as they actually felt quite healthy.

#### Patients Focus on What Works

Patients trusted their own strategies for dealing with their problems and lived a good life without feeling a need for more understanding of their illness. Several patients focused on what they knew worked for them considering their individual limitations. Sometimes patients put their illness to the test and challenged their symptoms, like when type 2 diabetes patients intentionally failed to maintain their prescribed diet (either by not eating or by eating sweets), often being surprised that the negative effects were not worse.

We ate a good dinner … and then tested our blood glucose level … after the dinner it was only 7.8-something … nothing particularly dangerous to get worked up about … so I took a bun and a cake with the coffee ... a second test showed that the levels had not changed much.

#### Patients have Confidence in Healthcare Providers

Patients did not believe that more understanding would make them feel better. Instead, they trusted in the healthcare providers' skills and knowledge of the illness, and in their ability to correctly assess the condition. Sometimes, patients placed themselves completely in the hands of the healthcare providers and even felt that the illness offered them certain benefits.

So I'll get a medical examination every year and meet NN [district nurse] once a year, even every six months … they take care of me … without the illness I wouldn't have gone to the healthcare centre.

Patients felt that it was essential to have continuity in terms of the healthcare providers met, in order to not have to repeat their medical history over and over again. It was particularly important that the healthcare provider possess expert knowledge of the patient's illness.

As soon as something comes up, I contact the doctor and ask what is happening and whether or not it is something to be concerned about.

## DISCUSSION

The results of this study show that patients vary in their degree of reflection about and understanding of their illness and that this reflection and understanding are related to their degree of trust in themselves or in others (see Table **3**). There are patients who have a great need for specific and deeper understanding of their illness and there are patients who have a more general rather than detailed need for understanding of their illness. There are also patients who have less need for understanding, who trust in their own ability to self-manage the illness or have confidence in the healthcare providers' expertise.

Some patients search for a deeper understanding of their illness and want detailed answers, ask many questions and want to know the causes of the illness. Such patients want to understand how the illness affects them and they do not simply accept that they had been diagnosed. They also have confidence in their own ability to influence their situation. Some patients try to understand the relationship between the root cause, the triggering factor and the present state when they search for understanding of the illness; these patients have trust in themselves. According to the “Illness perception” model [22], patients who are reflective in personal terms reflect more carefully and deeply about their illness, want to achieve deeper understanding of the illness, and search for causal explanations. Several studies have underlined that a patient's desire for more understanding of their illness is important for the recovery process [1-3].

Patients who rather reflect in general terms about their illness compare their test results and the medication effects of their illness without understanding the reference values. However, they have no need to understand the background of, for example, a test result or a blood glucose value, and they show that they have great confidence in the ability of the healthcare staff. They prefer to place their trust in others. The patients think in terms of preventing complications and are satisfied with a more general and not particularly detailed understanding of their situation.

Some patients are less reflective, put their trust in others, and thus accept and manage their life. Alsén *et al*. [22] model shows that patients who want healthcare providers to provide clear information about their illness are reflective in general terms. They do not reflect on deeper causes or effects and are satisfied with a general understanding of their illness situation. They put faith in the knowledge of healthcare providers and do not feel they that can improve their own situation. They do not need detailed information and do not have theories about the origin of their illness. They do not let the illness bother them more than absolutely necessary in their daily life. Dahlberg and Segesten [21] believe that the patient's perception of their health and illness situation is based on their individual life-world perspective. The life-world consists of, among other things, the patient's memories, everyday experiences, and expectations for the future. They do not believe that drugs or other therapy can help them and they develop their own strategies to deal with the illness.

When patients focus on what works for them they manifest trust in themselves. They test their way forward and find their own strategies to be able to live their life. They therefore do not want to get detailed information about their illness but rather distance themselves from it. These patients are less reflective about their illness; their reflections are more superficial and sporadic, especially when the symptoms do not affect them [22]. They also have more predetermined ideas and are more difficult to influence. They also place great trust in the healthcare providers' expertise [trust in others] and do not feel that they need to find out more on their own.

There seems to be a connection between reflection, understanding and how much illness symptoms affect the patient's daily life. For example, high blood pressure does not always show symptoms in everyday life and therefore the patient does not actively search for understanding in the same way as would a patient suffering from, for instance, constant and unbearable pain. Patients whose illness poses an existential threat are more likely to reflect more about their illness and what treatment methods are available. This agrees with Kleinman's [13] observation that one should consider the patient's self-perceived state of health [illness] and the medical explanation of the disorder [disease] as an interdependent whole, and with the insight that a patient's lack of reflection and understanding of their illness can be linked to the patient's level of understanding of the illness and interest in seeking information about the illness [1, 3]. The patients in the study used reflection to understand their illness in different ways, which also affected the learning process itself and how actively they used the understanding of the illness to manage it in everyday life. This confirms the importance, noted in several studies [8-11] of providing individualised information that takes the patient’s ability to understand the illness better into account.

### Limitations

A limitation of this study may be that the recruitment of the participants was carried out by the healthcare providers, possibly selecting individuals who were better than others at expressing their problems, which may have influenced the study's trustworthiness. However, there was variation in the depth and duration of the illness, abilities, and verbal ability among the participants, indicating that they were a heterogeneous group with varied understanding of the illness. Other limitations were that the participants’ average age was relatively high and that there were slightly fewer women than men, which may limit the transferability of the result to similar groups. Another limitation was that the study focuses only on patients with long-term illnesses, which may entail that its conclusions are only transferable to patients with similar conditions. Yet another limitation was that only Swedish-speaking people were interviewed, which may affect the transferability of the study regarding people with different ethnic or cultural backgrounds. Such aspects may be of interest for future studies.

## CONCLUSION

This study focuses on how the patients reflect on and understand their illness and how this understanding affects their trust in themselves or others. Patients reflect on their illness at different levels, from a personal level to a more general level, and have different needs and use of understanding of their illness. Some patients do not reflect very much at all about their illness and develop their own strategies to cope with it. There are variations regarding how the reflection is used depending on the patients' level of confidence in their own ability to manage the illness, as well as regarding their reliance on the skills of others, in particular healthcare providers. There is nothing that supports the idea that more understanding of the illness always helps patients manage their daily life. This depends on how the understanding uses as knowledge in action. Patients, who want to understand more about the illness, create their own understanding, trust themselves and could use the understanding as knowledge in action and thus better understand how to manage the illness. At the same time, there are patients who have a good ability to function without having the need to know so much about the illness and is content to focus on what works. They have found other, compensatory ways to manage the illness.

To enable and promote the use of understanding of the illness, the understanding provided should be tailored to the patients' needs, abilities and levels of health. The goal must always be that the patient should be able to trust in their own understanding and through this develop a better ability to manage their disease and symptoms in daily life (improved self-care ability). Special attention should be paid to patients who have a high level of trust in others in order to strengthen their trust in themselves. Clinical interventions with both patients and healthcare providers are necessary. Interventions may also contribute to the development of an assessment tool based on the “Illness perception” model in order to assess the patients' need for understanding and opportunities for managing their illness. It is also important to study other age groups (younger persons and children) and whether or not a patient's need for understanding of the illness, as well as perception of it, depends on the illness type and on for how long the patient has had the illness.

## Figures and Tables

**Table 1:** Sociodemographic characteristics of the participants (N=11).

**Variable**	**N=11 (Frequency %)**
**Age** Mean Range Median	68 49-81 69
**Sex** Male Female	7 (64%) 4 (36%)
**Marital status** Single Married/cohabitation	1 (9%) 10 (91%)
**Socioeconomic situation** Employed Retired On disability pension	3 (27%) 7 (64%) 1 (9%)
**Ethnicity** Born and raised in Sweden	11 (100%)

**Table 2:** Example of analysis process with sub-themes and themes.

**Original interview text**	**Condensation unit**	**Code**	**Subtheme**	**Theme**
And then there are the associations … I've been online and read some articles that they publish … I recognize some stuff and there is of course also a bit about the various reference values and such things … So I always ask how my values are so I have little control myself.	There are associations I've been online and read some articles that they publish	Associations Articles on the Internet Read about the reference values	Patients search for deeper understanding about their illness through various sources of information	**Patients actively seek knowledge** **of the disease for causal explanations**

**Table 3:** The result (themes and sub-themes) in relation to the Illness reasoning model.

**Illness reasoning**	**Reflective in personal** **terms**	**Trust in oneself**	**Trust in others**
	Patients actively seek knowledge of the disease for causal explanations	Patients understand their illness through various sources of information Patients try to understand the relationship between root cause, trigger factor and present state	Patients want clear information from healthcare providers to better understand the illness
	**Reflective in general** **terms**	**Trust in oneself**	**Trust in others**
	Patients compare different perspectives to get knowledge for preventing complication of their illness	Patients use experiences and information from other peoples’ illness situations and compare them with their own situation	Patients compare test results and reported medication effects without understanding the reference values
	**Less reflective**	**Trust in oneself**	**Trust in others**
	Patients have trust in healthcare providers knowledge and develop strategies to manage life	Patients accept and manage life Patients focus on what works	Patients have confidence in healthcare providers
